# The Role of Visual Feedback on Power Output During Intermittent Wingate Testing in Ice Hockey Players

**DOI:** 10.3390/sports6020032

**Published:** 2018-04-09

**Authors:** Petr Stastny, James J. Tufano, Jan Kregl, Miroslav Petr, Dusan Blazek, Michal Steffl, Robert Roczniok, Milos Fiala, Artur Golas, Piotr Zmijewski

**Affiliations:** 1 Faculty of Physical Education and Sport, Charles University, 16252 Prague, Czech Republic; tufano@ftvs.cuni.cz (J.J.T.); jan.kregl@rytirikladno.cz (J.K.); petr@ftvs.cuni.cz (M.P.); dusanmpk@seznam.cz (D.B.); steffl@ftvs.cuni.cz (M.S.); fiala@ftvs.cuni.cz (M.F.); 2 Department of Theory and Practice of Sport, The Jerzy Kukuczka Academy of Physical Education, 40-065 Katowice, Poland; r.roczniok@awf.katowice.pl (R.R.); a.golas@awf.katowice.pl (A.G.); 3 Faculty of Medicine, University of Information Technology and Management in Rzeszow, 235-225 Rzeszow, Poland; zmijewski@op.pl

**Keywords:** peak power, elite sport, anaerobic performance, intermittent load

## Abstract

**Background:** Visual feedback may help elicit peak performance during different types of strength and power testing, but its effect during the anaerobic Wingate test is unexplored. Therefore, the purpose of this study was to determine the effect of visual feedback on power output during a hockey-specific intermittent Wingate test (AnWT6x6) consisting of 6 stages of 6 s intervals with a 1:1 work-to-rest ratio. **Methods:** Thirty elite college-aged hockey players performed the AnWT6x6 with either constant (*n* = 15) visual feedback during all 6 stages (CVF) or restricted (*n* = 15) visual feedback (RVF) where feedback was shown only during the 2nd through 5th stages. **Results:** In the first stage, there were moderate-to-large effect sizes for absolute peak power (PP) output and PP relative to body mass and PP relative to fat-free mass. However, the remaining stages (2–6) displayed small or negligible effects. **Conclusions:** These data indicate that visual feedback may play a role in optimizing power output in a non-fatigued state (1st stage), but likely does not play a role in the presence of extreme neuromuscular fatigue (6th stage) during Wingate testing. To achieve the highest peak power, coaches and researchers could provide visual feedback during Wingate testing, as it may positively influence performance in the early stages of testing, but does not result in residual fatigue or negatively affect performance during subsequent stages.

## 1. Introduction

To monitor training adaptations and quantify peak performance capabilities, field- and laboratory-based testing should be conducted in a manner that is standardized, reliable and valid. To ensure testing validity and create a testing environment that encourages peak performance, testing procedures should include guidelines for warm-up, equipment selection, test familiarization, verbal instruction, and types of feedback. Among these considerations is the inclusion of visual feedback [1,2]. The visual feedback has been found important in testing short-term maximal effort to reach the highest isokinetic force output [2,3,4]; however, its influence on maximum force or power output in other kinds of tests is not sufficiently described.

Previous research has shown that the presence of visual feedback elicits greater performance compared to when visual feedback is absent during short-term maximal testing efforts that are characteristic of strength and power testing [3,5]. One such test is the anaerobic Wingate test (AnWT), which is commonly used to measure peak power output (PP) and anaerobic capacity in team sport athletes [6,7,8] such as ice hockey players [9,10,11]. Although the traditional AnWT includes a single 30 s continuous maximal effort sprint, a 6 s version of the AnWT can serve as a shorter alternative to the 30 s version [12,13]. Furthermore, the 20 s AnWT [14], 15 s AnWT [15,16] and 6 s AnWT [12,13] have been found to be valid measurements of PP and result in the same PP output as AnWT 30 s [13,14,16,17]. Considering that ice hockey includes repeated bouts of maximal effort and that players can sprint the length of an ice hockey rink in about 6 s [18,19,20] (transition between the defense and offense zone), an intermittent version of AnWT using multiple 6 s stages (AnWT6x6) may increase the ecological validity of the test and provide insight into a player’s ability to maintain intermittent maximal power output (i.e., sprint performance) during a typical on-ice shift that can last between 30 and 85 s [10]. The use of intermittent AnWT testing has already been applied among National Hockey League players, where a 30 s AnWT has been replaced by a 4 × 5 s test variation in 2005 [10]. 

Regardless of the duration of the AnWT, athletes exert themselves maximally, neuromuscular fatigue ensues, and performance decreases. As previous research indicates that visual feedback can influence power output during a single maximal effort in the absence of fatigue [3,5] and can positively affect performance during repetitive intermittent low-intensity muscle actions [21,22], visual feedback may also play a role in maximal intermittent tasks such as the AnWT6x6. However, it is unknown whether the presence of visual feedback plays a role in both, fatigued and non-fatigued states during maximal intermittent efforts. Therefore, the purpose of this study was to determine whether visual feedback could influence power output during an intermittent AnWT test. Since power output is largely related to neuromuscular excitation and mental focus [23,24], it was hypothesized that restricting visual feedback would negatively affect power output.

## 2. Materials and Methods

### 2.1. Experimental Approach to the Problem

The present cross-sectional study was performed in the Laboratory of Human Adaptation during a one-week break in the middle of a competitive ice hockey season. Subjects reported to the laboratory for a single session, beginning with anthropometric measurements and finishing with an AnWT6x6 test. In short, subjects were randomly assigned to one of two groups: either a group that received constant visual feedback (CVF) during all 6 stages of the intermittent AnWT6x6 test, or a group that received restricted visual feedback (RVF), receiving feedback only during the 2nd through 5th stages of the AnWT6x6. In this manner, it was possible to determine the effect of restricting visual feedback on power output during a non-fatigued (1st stage) and fatigued (6th stage) state during the intermittent AnWT6x6. The purpose of this design was to elicit a similar fatigue protocol in stages 2–5 before the last bout.

### 2.2. Subjects

All participants (*n* = 30) were college-aged male ice hockey players (18–25 years) who were members of the national academic ice hockey team pool in the Czech Republic (Table 1), which ensures the motivation for maximal performance output [25]. All subjects were periodically tested by means of AnWT 30 s using 10% of body weight load for at least four previous years, and performing the last AnWT test three months prior to the experiment. All subjects were healthy and did not have any acute or chronic injuries. The subjects did not perform any resistance or high-intensity exercise 72 h before testing, did not adhere to any low-carbohydrate or energy-restrictive diets, and did not use any stimulants before or during the test. Written informed consent was obtained from all subjects and the study was approved by the Institutional Ethics Committee at the Faculty of Physical Education and Sport at Charles University in accordance with the ethical standards of the Helsinki Declaration of 1983.

### 2.3. Procedures

After anthropometric measurements, participants performed a 5-min general warm-up (10 repetitions of 25 m sprints with progressively increasing speed) which has been recommended for the 6 s AnWT test [26]. Next, subjects performed 5 split squats on each leg, 5 full squats, and 5 bodyweight squat jumps, rested for 30 s, and repeated the process until 5 sets of the circuit were completed. The squats were included because they can increase power performance during the test [27,28]. Participants were then seated on the cycling ergometer and pedaled at a self-selected cadence without resistance for 2 to 3 min. Then, in line with previous research, participants were familiarized with the upcoming test start by administration of the test start and performing three pedal revolutions with resistance [29]. After three revolutions, the resistance was removed and the participant cycled without resistance for 30 s. Finally, the AnWT6x6 test was performed against resistance with maximal effort according to each subject’s assigned group (CVF or RVF).

### 2.4. Anthropometry

Body mass (BM) in kg and height in cm were assessed to calculate individualized constant braking resistance for the AnWT6x6. To estimate body fat percentage (BF) and fat-free mass (FFM), the skinfold (SK) thickness was measured at six sites (pectoral, subscapular, triceps, suprailiac, abdominal and thigh) by a trained technician using Harpenden Skinfold Calipers (Baty International, Burgess Hill, UK) and using a formula developed for strength athletes [30,31]: BF (%) = (pectoral SK) × 0.148 + (subscapular SK) × 0.075 + (triceps SK) × 0.077+ (suprailiac SK) × 0.16 + (abdominal SK) × 0.152 + (thigh SK) × 0.102.

### 2.5. 6 × 6s Anaerobic Wingate Test—AnWT6x6

The AnWT6x6 was conducted on a calibrated friction-loaded cycle ergometer (Monark 894E Peak bike, MONARK, Sweden) interfaced with a microcomputer. The cycle cranks were equipped with toe-clips to prevent the feet from slipping. To simulate the repeated sprint bouts that occur during an elite ice hockey shift [31,32], the AnWT6x6 test included six individual 6-s maximal-intensity stages with 6 s rest intervals (i.e., pedaling without resistance), resulting in a 1:1 work-to-rest ratio [32]. The test began from a seated rolling start and the constant fiction-loaded resistance (100 g·kg^−1^) [33] was added as soon as participants exceeded 120 revolutions·min^−1^. Subjects were instructed to stand as soon as they felt resistance [34,35] and verbal encouragement was provided during all active stages of the test [36,37]. After 6 s of maximal pedaling against resistance, the resistance was removed and subjects returned to a seated position and resumed a 120 revolutions·min^−1^ cadence. After 6 s of unloaded cycling during the rest period, resistance was again added to the ergometer and the process was repeated until the final stage was complete.

In each 6-s stage, power output was calculated using a rolling average every 1 s, and the 1 s with the greatest value was used as PP. The absolute PP values were expressed in Watts (W), PP relative to BM (PPkg) in W·kg^−1^, and PP relative to FFM (PPFFM) in W·kgFFM^−1^ [38]. Additionally, the power decrement was calculated as the percentage decrease from the 1st stage to the 6th stage for PP (PPDec), PPkg (PPkgDec) and PPFFM (PPFFMDec).

### 2.6. Visual Feedback

**Figure 1:**
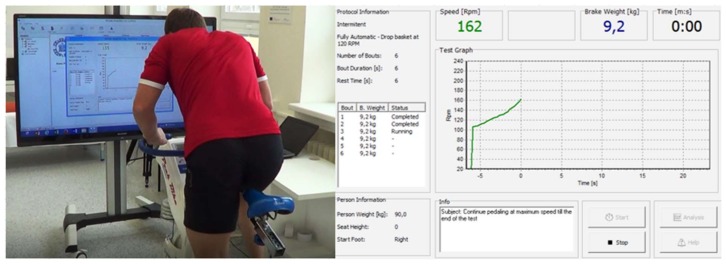
Testing configuration with visual feedback appearing 2 meters in front of the cycle ergometer. The participants were instructed to look at the revolution-time curve, which was adjusted to their field of vision during specific warm-up.

A 50-inch television (AQUOS, 1.35 m diagonal, Sharp Corporation, Osaka, Japan) was connected to a computer, placed 2 m in front of the ergometer’s handlebars (Figure 1), and remained turned on for the CVF group, providing subjects with a full view of the revolution-time curve in addition to numerical revolutions·min^−1^, which represents the power output curve. The screen was readjusted to the participant’s visual field during the initial test start administration. The RVF group received visual feedback only during the 2nd, 3rd, 4th and 5th stages. For the bouts with visual feedback, the participants were instructed to look at the revolution-time curve when they accelerated to start the test and during the 6 s bout. The person giving verbal encouragement controlled the status of visual feedback and provided additional instructions if visual feedback was interrupted. 

### 2.7. Statistical Analyses

All statistical analyses were performed with STATISTICA version 12 (StatSoft, Inc., Tulsa, OK, USA) with α = 0.05. Data normality was tested using the Shapiro–Wilk test (Table 2). To determine whether interactions or main effects were present for PP, PPkg and PPFFM, individual 2 × 6 (group × AnWT6x6 stage) analyses of variance (ANOVA) were performed and followed up with Tukey´s post-hoc tests. The *t*-Test was used to determine differences in anthropometrical measures between groups (Table 1) and PP output in the 1st and 6th stages of the AnWT6x6. Additionally, effect sizes were calculated (Cohen’s *d*) and can be interpreted as small (*d* = 0.20 to 0.49), moderate (*d* = 0.50 to 0.79), and large (*d* ≥ 0.80) [39].

## 3. Results

**Figure 2:**
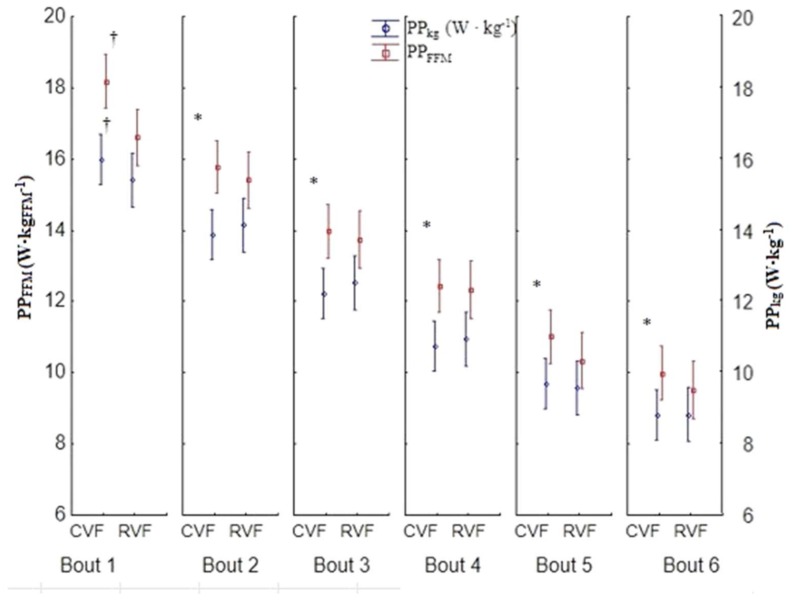
The relative power output during each bout of Anaerobic Wingate test with constant and restricted visual feedback (mean ± SD). CVF = constant visual feedback group, RVF = restricted visual feedback, PP_kg_ = peak power relative to body mass, PP_FFM_ = peak power relative to fat-free mass. ^†^ significant difference in PP_FFM_ between protocols within the same stage, * significant difference for both variables between stages, collapsed across protocol.

**Figure 3:**
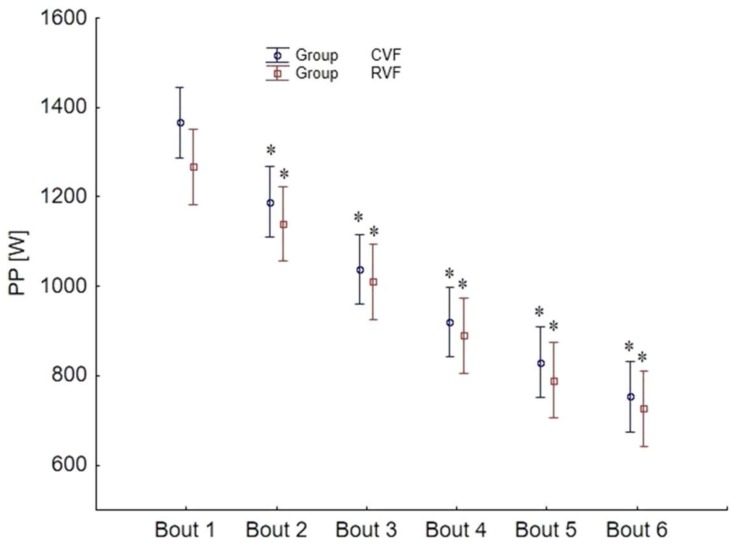
The absolute power output during each bout of Anaerobic Wingate test with constant and restricted visual feedback (mean ± SD). CVF = constant visual feedback, RVF = restricted visual feedback, * significantly different from previous stage.

There were no differences in anthropometric measures between groups (*p* > 0.05, Table 1), and all data were normally distributed (W range between 0.89 and 0.98). The *t*-tests showed significant differences with a large effect size between groups for PPFFM (*t* = −2.95, *p* = 0.007, Cohen *d* = 1.10, *r* = 0.48) during the first stage (Figure 2), but no other variables were different between groups during the 1st or 6th stage. The ANOVA revealed no group by stage interactions, but there were individual main effects for time (stage) for PP (F_5,135_ = 242, *p* < 0.001, η^2^ = 0.89, power α = 0.99), PPkg (F_5,135_ = 233, *p* < 0.001, η^2^ = 0.89, power α = 0.99), and PPFFM (F_5,135_ = 228, *p* < 0.001, η^2^ = 0.89, power α = 0.99) (Figure 2 and Figure 3). Follow-up tests revealed differences for all variables between all six stages (Figure 2 and Figure 3), with power output decreasing as the test progressed. The power decrement did not significantly differ between both groups, and PPDec was 45.40 ± 7.85%, PPkgDec was 45.38 ± 7.91%, and PPFFMDec was 45.00 ± 8.09% in values combined from both groups.

## 4. Discussion

The main finding of this study is that the presence of visual feedback likely affected power output in the initial stage of the intermittent AnWT6x6, but did not affect power output when subjects were already fatigued during the final stage. The middle stages of the test (i.e., stages 2 through 5, where both groups had visual feedback) resulted in similar power outputs for both conditions, indicating that the greater power output in the 1st stage of CVF did not result in accumulated residual fatigue later during the test. Therefore, it may be warranted to include constant visual feedback during the intermittent AnWT6x6 if aiming to determine peak power output in hockey players.

Firstly, it cannot be determined whether visual feedback during the initial stage “increased” performance or if the absence of visual feedback “decreased” performance. The nature of the experimental design only allows for the conclusion that the presence of visual feedback had a moderate-to-large effect on power output during the initial stage of the AnWT6x6. This result is in agreement with previous findings [3,5,40], where visual feedback resulted in greater peak power output compared to no visual feedback. In summary, the isokinetic knee flexion and extension [3,40], leg press [5] and AnWT testing should be provided with visual feedback to ensure greatest force or power output. This testing consistency might be justified by previously reported association between power output observed between AnWT and isokinetic knee flexion/extension (*r* = 0.71 to 0.86) [41,42]. Therefore, it seems to be appropriate that tests with similar output are sensitive for the same kind of test condition (i.e., presence of visual feedback). 

Although the data of the present study indicate that visual feedback may play a role in the beginning of the AnWT6x6, power output during the final stage was not different between protocols and effect sizes were negligible to small (compared to the moderate-to-large effect during the initial stage). Therefore, it can be concluded that the presence of visual feedback may not play a role in the presence of central and peripheral fatigue [43]. Unfortunately, the purpose of this study was to determine whether visual feedback affects AnWT performance, not to investigate the mechanisms of the effect. However, future studies may aim to investigate the relationship between central and peripheral fatigue and the ability of visual feedback to affect performance.

The peak power output relative to BM in our participants (PPkg = 15.82 ± 1.45 W·kg^−1^) was from about 15% [44,45] to roughly 30% greater [11] than those reported in previous studies. It is possible that greater relative PP values occurred due to a greater resistance used in the present study (10% of BM compared to 7.5%) [11,44,45]. Additionally, it has been shown that performing the AnWT in a standing position results in greater power outputs [35], indicating that the standing position may have played a role in the greater power outputs of the present study compared to other studies where subjects were not standing [35]. A final possible explanation for the greater power outputs in the present study may be that PP was calculated using a rolling 1 s window, which may provide greater PP values than methods utilizing longer timeframes such as 5 s [11,44,45]. Although absolute and relative PP values were greater in our subjects, the absolute and relative power decrements were similar to previous studies [46].

The PPDec, PPkgDec and PPFFMDec of about 45% in our subjects using the AnWT6x6 is similar to the power decrement reported in previous studies using the traditional AnWT 30 s protocol (49%) [44,46]. Although the AnWT is often used to determine PP output, such a basic measure can be recorded in as few as 6 s [12,13]. In ice hockey, PP may be important, but the ability to repeatedly perform movements with maximal power and short recovery times (i.e., multiple sprints while on the ice) [32] may be of greater importance. As this is the first study to our knowledge to use an intermittent AnWT, it can be used as a benchmark for subsequent intermittent AnWT tests, showing that intermittent Wingate testing may have a similar decrease power output compared to a single 30 s bout, but can provide additional information regarding a player’s intermittent capacity.

Visual feedback might be used to improve exercise technique [47] in runners or to increase performance in the cycling aerobic test [48] in the untrained population. The results of the present study suggest that visual feedback may increase anaerobic performance in well-trained male athletes who are highly familiarized with the test protocol. Therefore, the application of visual feedback is recommended for various tests in various populations.

## 5. Conclusions

Visual feedback during AnWT can enhance power output in the initial test bout. The visual feedback is affected by fatigue with no power output enhancement during the final bout of AnWT6x6. Additionally, visual feedback should be provided during all stages of AnWT testing in order to elicit peak performance. Therefore, it is recommended to use the AnWT6x6 with visual feedback to provide coaches with information regarding a player’s ability to repeatedly produce maximal power output for the duration of a typical ice hockey shift.

## Figures and Tables

**Table 1:** Participant characteristics.

	Constant Visual Feedback (*n* = 15)	Restricted Visual Feedback (*n* = 15)	*t*-Test (*p* Value)	Combined (*n* = 30)
Age (years)	22.86 ± 2.25	21.96 ± 2.05	0.81	22.45 ± 2.11
Height (cm)	186.92 ± 4.78	186.0 ± 4.62	0.63	186.5 ± 4.85
Body mass (kg)	82.23 ± 8.94	85.26 ± 8.99	0.37	83.8 ± 8.9
Fat mass (%)	9.80 ± 5.45	9.33 ± 4.90	0.33	9.69 ± 5.11
Fat-free mass (kg)	76.17 ± 7.20	74.89 ± 5.93	0.60	75.5 ± 6.5
Playing experience (years)	15.64 ± 1.51	15.02 ± 1.35	0.67	15.22 ± 1.45

**Table 2:** Power output during 6 × 6 Anaerobic Wingate Test.

Stage	Parameter	CVF (Mean ± SD)	RVF (Mean ± SD)	Effect Size (Cohen’s *d*)	Together (Mean ± SD)
1	PP (W)	1379 ± 150	1266 ± 187	0.67	1324 ± 175
	PPkg (W·kg^−1^)	16.2 ± 1.34	15.41 ± 1.50	0.56	15.82 ± 1.45
	PPFFM(W·kg^−1^)	18.43 ± 1.62	16.61 ± 1.68	1.10	17.56 ± 1.88
2	PP (W)	1191 ± 159	1139 ± 186	0.30	1167 ± 171
	PPkg (W·kg^−1^)	13.98 ± 1.27	13.94 ± 1.64	0.03	13.96 ± 1.43
	PPFFM(W·kg^−1^)	15.89 ± 1.31	15.51 ± 1.76	0.24	15.71 ± 1.53
3	PP (W)	1040 ± 148	1010 ± 187	0.18	1025 ± 165
	PPkg (W·kg^−1^)	12.21 ± 1.15	12.49 ± 1.73	0.20	12.35 ± 1.44
	PPFFM(W·kg^−1^)	13.87 ± 1.43	13.80 ± 1.94	0.04	13.84 ±1.67
4	PP (W)	926 ± 154	889 ± 178	0.22	908 ± 164
	PPkg (W·kg^−1^)	10.87 ± 1.52	10.94 ± 1.52	0.05	10.90 ± 1.49
	PPFFM(W·kg^−1^)	12.34 ± 1.57	12.19 ± 1.68	0.09	12.27 ± 1.60
5	PP (W)	831 ± 152	790 ± 143	0.28	811 ± 147
	PPkg (W·kg^−1^)	9.74 ± 1.49	9.80 ± 1.17	0.04	9.77 ± 1.32
	PPFFM(W·kg^−1^)	11.07 ± 1.68	10.98 ± 1.54	0.06	11.03 ± 1.58
6	PP (W)	756 ± 139	726 ± 124	0.23	741 ± 130
	PPkg (W·kg^−1^)	8.88 ± 1.43	8.82 ± 1.03	0.05	8.85 ± 1.23
	PPFFM(W·kg^−1^)	10.07 ± 1.50	9.66 ± 1.27	0.30	9.87 ± 1.38

Peak power output was obtained from the best 1 s interval, PP = peak power, PPkg = peak power per kg body mass, PPFFM = peak power per kg fat-free mass, SW = Shapiro–Wilk test; CVF = constant visual feedback, RVF = restricted visual feedback.
